# The oncogenic signalosome: SQSTM1/p62 as a master integrator of signaling, metabolism, and autophagy in cancer

**DOI:** 10.1007/s43188-026-00349-9

**Published:** 2026-03-30

**Authors:** Eun-Ji Choi, Sang-Min Jeon

**Affiliations:** 1 iDeTOX LAB, College of Pharmacy, and Research Institute of Pharmaceutical Sciences, Seoul National University, Seoul, 08826 Korea; 2 Natural Products Research Institute, College of Pharmacy, Seoul National University, Seoul, 08826 Korea

**Keywords:** SQSTM1/P62, NFE2L2/NRF2, AMPK, Signaling, Metabolism, Cancer

## Abstract

Sequestosome 1 (SQSTM1/p62), long established as a selective autophagy receptor and ubiquitin-binding scaffold, is now recognized as an emerging regulatory hub that integrates signaling, metabolism, and stress adaptation in cancer. Beyond its canonical role in proteostatic cargo degradation, recent advances have revealed that p62 orchestrates nutrient sensing, redox control, innate immune signaling, and metabolic reprogramming through highly dynamic, context-dependent mechanisms. A nascent paradigm emerging from recent studies is that p62 function is specified by a hierarchical post-translational modification (PTM) code, with phosphorylation acting as the primary regulatory layer. Site-specific phosphorylation events—together with modulatory PTMs such as S-acylation, arginine methylation, and O-GlcNAcylation—reshape p62 interaction networks, liquid–liquid phase separation (LLPS) behavior, and signaling output. Through these mechanisms, p62 operates as a sophisticated signal–metabolism interface that couples stress signaling pathways, including NFE2L2/NRF2, AMPK, mTORC1, and NF-κB to the systemic rewiring of glucose, lipid, amino acid, and nucleotide metabolism. Notably, a phosphorylation-dependent positive feedback loop between p62 and AMPK has emerged as a key driver of metabolic plasticity, enabling tumor cells to survive and proliferate under the stringent metabolic stress conditions of the tumor microenvironment. This review integrates recent mechanistic insights into the PTMs, phase behavior, and signaling hub functions of p62, highlighting how these principles manifest in distinct oncogenic contexts, such as lung, prostate, and brain tumors. We further discuss emerging therapeutic strategies that seek to modulate p62-centered assemblies rather than indiscriminately inhibit p62 function. Collectively, these findings position p62 as a phosphorylation-governed oncogenic nexus whose precise manipulation may enable new strategies for context-dependent precision oncology.

## Introduction

The cellular proteostasis network and signal transduction pathways were long viewed as distinct functional dichotomies: one dedicated to waste disposal and quality control, the other to environmental adaptation and cellular decision-making. The discovery of Sequestosome 1 (SQSTM1/p62) transcended this boundary, revealing a molecular bridge between protein degradation and survival signaling. First described in 1995 as a 62-kDa SH2-binding partner of p56lck [1], p62 is now recognized as a ubiquitously expressed, highly conserved adaptor that coordinates multi-faceted signal transductions and stress responses. Work in the mid-1990s rapidly expanded p62’s functional identity. Its ability to concentrate ubiquitinated proteins into inclusion-like structures led to the term “sequestosome,” [2] and the oxidative stress–inducible transcript A170 was later shown to encode the murine ortholog of p62 [3, 4]. Mechanistically, the identification of a C-terminal Ubiquitin-Associated (UBA) domain [5–7] together with an LC3-Interacting Region (LIR) [8, 9] established p62 as the prototypical selective autophagy receptor. p62 recognizes ubiquitinated cargo and delivers it to LC3/GABARAP-positive autophagosomes for lysosomal degradation. [8, 10–13]

In cancer, p62 exhibits profound and nuanced context dependence. In normal tissues, p62-mediated selective autophagy exerts a tumor-suppressive effect by limiting the accumulation of aggregates and dysfunctional organelles that fuel reactive oxygen species (ROS) and genome instability [14, 15]. In many established tumors, however, p62 is aberrantly elevated and often associates with poor prognosis, including hepatocellular carcinoma, pancreatic ductal adenocarcinoma, breast cancer, prostate cancer, and glioblastoma [16–20]. Notably, p62 accumulation is not merely a passive marker of impaired autophagic flux [21]. When abundant, p62 can function as a dynamic scaffold that integrates signaling and metabolic regulators, thereby reshaping stress adaptation.

This hub-like activity is enabled by p62’s multidomain, multivalent architecture. p62 couples proteostasis to nutrient and energy sensing (for example, mTORC1 and AMPK) [22–25], oxidative stress control (KEAP1–NFE2L2/NRF2), and inflammatory signaling (TRAF6–NF-κB) [15, 26–28]. Recent studies add a further layer of architectural organization: p62 can assemble higher-order structures, including biomolecular condensates [29, 30], and its outputs are tuned by post-translational modifications that shift the balance between cargo clearance and signal propagation [31, 32].

In this review, we summarize the historical foundations and core mechanisms of p62 biology and examine how elevated p62 supports tumor progression. We highlight emerging principles—condensate-based organization, metabolic feedback wiring, and therapeutic strategies aimed at disrupting p62-centered network behaviors—that are reshaping how p62 is conceptualized as a targetable node in cancer.

## Protein structure and liquid–liquid phase separation (LLPS)

**Fig. 1:**
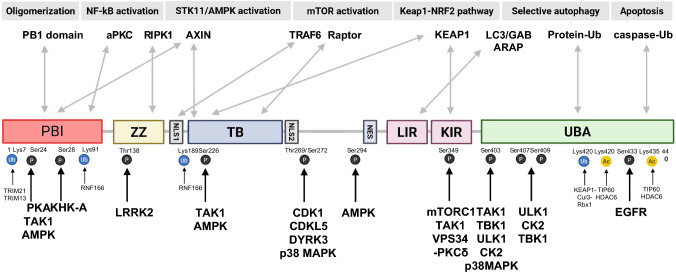
Structural architecture and phase transition dynamics of SQSTM1/p62. The schematic illustrates the multivalent domain architecture of p62 and its capacity for higher-order assembly, highlighting how conserved modules—including the N-terminal PB1 domain, ZZ domain, TB domain, LIR motif, KIR motif, and C-terminal UBA domain—coordinate to drive liquid–liquid phase separation (LLPS). Synergistic interactions between PB1-mediated head-to-tail polymerization and UBA-mediated crosslinking of polyubiquitinated substrates facilitate the transition from a diffuse cytosolic state to condensed p62 bodies (sequestosomes or aggresome-like induced structures [ALIS]). These biomolecular condensates function as biochemical reaction crucibles that concentrate specific signaling and metabolic regulators, thereby providing a specialized microenvironment to orchestrate stress-adaptive cellular outputs

P62’s functional breadth is encoded within its modular domain architecture and its capacity to undergo phase transitions. Rather than acting as a simple binding protein, p62 serves as a scaffold that concentrates ubiquitin signals and selected partners into shared microcompartments, thereby organizing biochemical reactions that would be stochastically inefficient by diffusion alone (Fig. 1).

### Domain architecture

Human p62 (~ 440 aa) is composed of conserved modules with distinct interaction functions. The PB1 domain (1–102) drives head-to-tail oligomerization and higher-order assembly, engaging other PB1 proteins (e.g., NBR1, MEKK3, PKCζ) to couple p62 to signaling modules [22]. The ZZ domain (122–167) provides a regulatory interface for cell-death and inflammatory circuits, including RIPK1 binding, and has been explored as a potential small-molecule–addressable pocket [33, 34]. The TB domain (225–251) binds TRAF6 to support K63-linked ubiquitin signaling and downstream NF-κB activation [24, 26, 35, 36]. The LIR motif (332–343) mediates docking to LC3/GABARAP proteins, with flanking phosphorylation tuning its binding affinity [8, 33, 37, 38]. The KIR motif (346–359) captures KEAP1 to modulate NRF2 signaling [39]. Finally, the UBA domain (386–440) binds polyubiquitin chains and helps define cargo selectivity, which is dynamically tuned by modifications such as Ser403 phosphorylation [40, 41].

### LLPS and p62 bodies

P62 is now considered a canonical LLPS-capable protein [42]. Instead of remaining diffusely distributed, p62 condenses into "p62 bodies" (also termed sequestosomes or aggresome-like induced structures [ALIS]) under stress or elevated ubiquitin load. Condensate formation is driven by molecular multivalency: PB1-mediated oligomerization builds the p62 scaffold, while UBA–ubiquitin interactions provide inter-molecular crosslinking. Once p62 and ubiquitinated cargo exceed a threshold concentration, the system undergoes liquid–liquid demixing. These condensates function as biochemical reaction crucibles, increasing local enzyme–substrate concentrations and accelerating pathway kinetics.

p62 has been demonstrated to undergo liquid–liquid phase separation (LLPS) both in vitro and in vivo through characteristic liquid-like behaviors including rapid FRAP recovery, droplet fusion, and 1,6-hexanediol sensitivity [30, 43]. LLPS spatially concentrates p62 and binding partners (LC3, ubiquitinated cargo, KEAP1) into membrane-less compartments, enhancing local concentrations and promoting selective autophagy and antioxidant signaling. However, a critical distinction exists between demonstrating that p62 can undergo phase separation (firmly established) and proving that LLPS material properties—liquid-like dynamics, interfacial tension, or selective permeability—are mechanistically required for biological function (still unresolved). Whether these functions depend specifically on phase separation physics versus simple spatial concentration through protein–protein interactions remains unclear. Establishing causation requires separation-of-function mutants: p62 variants retaining binding competency but lacking phase separation capacity. If such mutants fail to support autophagy or NRF2 activation despite maintained interactions, this would prove condensate properties are functionally indispensable. However, systematic characterization of such mutants has not been reported for p62. Recent technical advances including calibrated half-FRAP methods [44] that quantify interfacial energies provide tools to distinguish genuine LLPS from alternative clustering mechanisms and rigorously test whether condensate material properties actively drive p62 functions.

### Post-translational modifications (PTMs) as output selectors

P62 interactions and condensate behavior are further tuned by post-translational modifications (PTMs). Accumulating evidence suggests that PTMs can act as output selectors, biasing p62 toward cargo degradation (selective autophagy) versus stress-adaptive signaling [32, 41]. The next section focuses on how this PTM “code” rewires binding networks and phase behavior, and how these changes intersect with cancer metabolism and therapy resistance (Fig. 1) [33].

## Post-translational modifications of p62

### Phosphorylation: the primary regulatory code

Phosphorylation is the most extensively studied and functionally decisive PTM of p62 [45]. While multiple studies support the concept that site-specific phosphorylation patterns shape distinct functional states of p62, the notion of a fully coordinated 'hierarchical PTM code' remains, in part, a working model rather than a universally established framework [46]. Well-characterized modifications such as Ser349 and Ser403 phosphorylation serve as anchor points for this model [31, 41], while emerging sites including Ser24 and Ser226 are increasingly recognized as functionally important but require further structural and functional validation to define their precise roles within the regulatory hierarchy [47, 48]. Rather than acting as isolated molecular events, site-specific phosphorylation patterns encode discrete p62 states that determine whether the protein primarily routes cargo toward degradation, stabilizes stress-adaptive signaling, or integrates both outputs into a coherent survival response (Table 1).

**Table 1:** Phosphorylation of p62: summary of "Phosphorylation: the primary regulatory code"

Phosphosite (domain)	Major kinase(s)	Structural/molecular effect on p62	Primary functional output	Dominant metabolic/signaling axis	Cancer or disease contexts highlighted	Experimental system (cell/model)	Metabolic or stress condition	Validation approach	References
Ser403 (UBA)	p38δ MAPK	Promotes phosphorylation under proteasomal stress	Aggresome targeting	Stress-adaptive proteostasis	Proteotoxic stress–adapted tumors	Cancer cell lines	Proteasome inhibition	Kinase inhibition; phospho-assay	Zhang et al., *J Cell Sci* 2018 [52]
	TBK1	Required for aggregate clearance; impaired in ALS variants	Selective autophagy	Proteostasis–neuro axis	ALS; neuro parallels in cancer	Neuronal models	Aggregate accumulation	TBK1 mutant analysis	Pilli et al., *Immunity* 2012 [40]; Foster et al., *MCN* 2020 [51]
	ULK1	Destabilizes UBA dimerization → ↑ polyUb affinity; promotes p62 body assembly	Enhanced aggrephagy / mitophagy	Proteostasis; stress buffering	Solid tumors (HCC, PDAC, NSCLC, breast)	HEK293; multiple cancer lines	Proteotoxic stress	S403A mutant; ULK1 overexpression	Matsumoto et al., *Mol Cell* 2011 [41]; Lim et al., *PLoS Genet* 2015 [49]
Ser407/Ser409 (UBA-proximal)	ULK1, CK2, TBK1	Fine-tunes UBA conformation	Cargo selection vs signaling bias	Autophagy–signaling balance	Therapy-stressed tumors (emerging evidence)	Cancer lines	Autophagy-inducing stress	Phosphomimetic mutants	Wirth et al., *Nat Commun* 2019 [37]
Ser349 (KIR)	VPS34–PKCδ axis	Reinforces S349 phosphorylation	Enhanced NRF2 output	Redox buffering	Aggressive tumors	Cancer cell lines	Growth factor input	Kinase inhibition	Xu et al., *Sci Adv* 2019 [53]
	mTORC1, TAK1	Mimics NRF2 ETGE; high-affinity KEAP1 binding	NRF2 activation; antioxidant signaling	PPP (NADPH), GSH; ferroptosis resistance	HCC, PDAC, ccRCC, KEAP1-mutant cancers	HCC cells; mouse liver	Oxidative/metabolic stress	S349A mutant; KEAP1 binding assay; KO mouse	Ichimura et al., *Mol Cell* 2013 [31]; Umemura et al., *Cancer Cell* 2016 [18]
Ser351 (mouse)	mTORC1	NRF2–mTORC1 feed-forward activation	Sustained antioxidant signaling	Immunometabolic fitness	Tumor-infiltrating CD8⁺ T cells	Mouse TIL models	Metformin; metabolic activation	Knock-in mouse	Nishida et al., *J Immunother Cancer* 2021 [54]
Ser28 (N-terminus)	Fructokinase A	Promotes oligomerization and KEAP1 sequestration	Antioxidant specification	NRF2-driven redox metabolism	Metabolic reprogramming	HCC cells	Fructose metabolism	Kinase inhibition	Xu et al., *Sci Adv* 2019 [53]
Ser24 (PB1)	PKA (basal); TAK1 (stress)	Regulates PB1-mediated oligomerization	Switch to signaling scaffold	AMPK activation	LKB1-deficient NSCLC	NSCLC cells; xenograft	Glucose starvation	S24A mutant; CRISPR deletion	Choi et al., *Autophagy* 2024 [47]
Ser226 (central)	TAK1	Enables lysosomal scaffolding	LKB1–AXIN complex assembly	AMPK-driven stress adaptation	NSCLC (STK11 loss)	Lung cancer cells; xenograft	Glucose deprivation	S226A mutant; in vivo growth assay	
Thr269/Ser272	CDK1	Stabilizes p62 during mitosis	Supports proliferation	Cell cycle–metabolism linkage	GBM; breast; colorectal	Cancer cell lines	Mitotic phase	Phospho-null mutant	Linares et al., *MCB* 2011 [57]
Thr269	DYRK3	Enhances TRAF6 binding; mTORC1 activation	Growth-promoting state	mTORC1 anabolism	Melanoma	Melanoma cells	Growth signaling	DYRK3 inhibition	Lee et al., *JBC* 2024 [58]
Thr138 (ZZ)	LRRK2	Alters signaling bias	Neuronal toxicity	Stress signaling	Parkinson’s disease	Neuronal models	Oxidative stress	Kinase mutant	Kalogeropulou et al., *Biochem J* 2018 [59]

The gatekeeper of p62’s degradative capacity resides within the UBA domain at Ser403 (S403) [41]. Phosphorylated by a suite of stress-responsive kinases—including ULK1, TBK1, TAK1, and CK2—markedly enhances the affinity of p62 for polyubiquitin chains by destabilizing UBA-mediated dimerization, thereby promoting p62 body assembly and selective sequestration of ubiquitinated cargo [25, 40, 41, 49]. ULK1-driven S403 phosphorylation is induced by proteotoxic stress to facilitate aggrephagy [49], while TBK1-dependent phosphorylation of p62 is required for efficient clearance of pathogenic protein aggregates and is impaired in ALS-associated TBK1 variants [50, 51]. Additional modulation of this pathway is provided by p38δ MAPK, which phosphorylates p62 to promote aggresome biogenesis under proteasomal stress [52]. Collectively, these phosphorylation events establish the degradative competence of p62 required for proteostasis maintenance. In the context of cancer, the elevation of Ser403 phosphorylation allows proliferating cells to buffer the increased burden of misfolded proteins and damaged organelles, thereby selectively reshaping proteome quality control to support tumor viability [25, 32, 40, 41, 49].

While Ser403 licenses ubiquitin decoding, phosphorylation of Ser349 (S349) within the KIR motif functions as a decisive switch for antioxidant signaling [31]. Through structural mimicry of the acidic ETGE motif found in NRF2, phosphorylated Ser349 enables p62 to bind KEAP1 with a higher affinity than NRF2 itself, leading to sustained NRF2 stabilization and activation [31]. This modification is promoted by oncogenic kinase inputs, including VPS34-driven PKCδ activity, and is further reinforced by non-canonical kinases such as fructokinase A (KHK-A), which directly phosphorylates p62 to enhance its oligomerization and KEAP1 sequestration capacity [53]. This constitutive engagement of the KEAP1–NRF2 axis is a hallmark of hepatocellular carcinoma and various solid tumors. By promoting robust redox buffering and ferroptosis resistance, Ser349 phosphorylation also operates in immune-metabolic contexts, where mitochondrial ROS and metformin signaling activate an NRF2–mTORC1–p62 phosphorylation loop in tumor-infiltrating CD8⁺ T lymphocytes [54].

Beyond the UBA–KIR axis, phosphorylation events in the N-terminal and central regions diversify p62 functionality by coupling it to assembly control, energy sensing, and cell-cycle regulation. Basal phosphorylation at Ser24, mediated by PKA, restrains p62 oligomerization [55], whereas stress-induced phosphorylation at Ser24 and Ser226, mediated by kinases such as TAK1, primes p62 to function as a signaling scaffold during metabolic stress. As discussed in "AMPK signaling: the double-positive feedback loop", these phosphorylation events are required for p62 to recruit and organize the STK11/LKB1–AXIN complex at the lysosome, positioning p62 directly upstream of AMPK activation [47]. Consistently, AMPK itself phosphorylates p62 to induce mitophagy and autophagic cell death programs in neural stem cells, highlighting reciprocal coupling between p62 phosphorylation and cellular energy sensing [56].

Additionally, Phosphorylation of p62 further extends into cell-cycle and growth control. During mitosis, CDK1 phosphorylates p62 at Thr269 and Ser272, ensuring timely mitotic progression and supporting tumor cell proliferation [57]. The same Thr269 residue is targeted by DYRK3, which enhances p62-dependent mTORC1 activation and promotes melanoma progression [58]. In parallel, disease- and tissue-specific kinases further diversify the p62 phospho-code: LRRK2 phosphorylates p62 to potentiate neuronal toxicity [59], while CDKL5-dependent phosphorylation programs regulate p62-mediated selective autophagy during antiviral responses in neural cells [60]. Growth factor signaling also converges on this axis, as EGFR-dependent phosphorylation events associated with p62 binding favor signaling-dominant states, and pharmacologic disruption of the EGFR–p62 interaction restores autophagic flux while suppressing oncogenic EGFR signaling in lung cancer models [61]. Collectively, these phosphorylation events establish a sophisticated regulatory hierarchy that defines p62 functional identity as a degradative receptor, a signaling scaffold, or an integrated stress coordinator.

### Ubiquitination: feedback modulation of p62 fate

P62 operates as both a perceptive reader and a covalent substrate of ubiquitin signaling. Ubiquitination serves as a secondary regulatory layer that tunes the stability and persistence of p62 assemblies rather than dictating their primary functional identity. Specifically, ubiquitination within the UBA domain—notably at Lys420—imposes a homeostatic constraint by restricting UBA homodimerization and subsequent ubiquitin binding, thereby acting as a negative feedback mechanism to prevent excessive, uncontrolled condensate growth [62, 63]. Conversely, K63-linked polyubiquitination by E3 ligases such as TRIM38 or TRAF6 stabilizes the p62 scaffold and sterically or functionally suppresses LC3 engagement [24, 35, 64]. This modification biases p62 toward signaling-competent conformers over degradative routing, a shift that facilitates sustained oncogenic signaling and drives tumor progression in specific malignant contexts.

### Lipid-linked modification: S-acylation as a spatial organizer

The S-acylation (palmitoylation) of p62, mediated by the acyltransferase ZDHHC19, functions as a critical spatial refinement mechanism. Covalent modification at Cys289 and Cys290 significantly increases the affinity of p62 condensates for autophagic membranes, orchestrating their targeted docking and subsequent engulfment by the nascent phagophore. The loss of this lipidation-dependent targeting precipitates the accumulation of enlarged, non-degradable p62 aggregates, effectively decoupling cargo sequestration from lysosomal execution [65]. This modification directly links lipid metabolic status to the physical execution of selective autophagy, suggesting that p62 serves as a sensor of membrane-lipid availability during proteotoxic stress.

### Arginine methylation: reinforcing condensate stability and redox signaling

PRMT6-mediated arginine methylation represents a biophysical reinforcement layer for p62 behavior. This modification enhances the liquid–liquid phase separation (LLPS) of p62, effectively strengthening the sequestration of KEAP1 within p62 condensates. Pharmacological or genetic inhibition of PRMT6-mediated methylation destabilizes these assemblies, thereby restoring KEAP1-mediated turnover of NRF2 and sensitizing cancer cells to ferroptosis [66]. This positioning highlights arginine methylation as a modulatory layer that reinforces the phosphorylation-defined, stress-adaptive states required for robust redox defense in aggressive tumors.

### O-GlcNAcylation: metabolic tuning of p62 behavior

O-GlcNAcylation couples flux through the hexosamine biosynthetic pathway (HBP)—and thus the availability of glucose and glutamine—to p62 functional output. By modulating both p62 phosphorylation patterns and condensate dynamics, O-GlcNAcylation acts as a nutrient-driven rheostat [67]. In nutrient-replete tumor microenvironments, high levels of O-GlcNAcylation bias p62 toward signaling-dominant states while simultaneously attenuating its autophagic turnover [68, 69]. This metabolic tuning reinforces the "metabolic privilege" of cancer cells, allowing them to prioritize growth-promoting signaling over homeostatic degradation when resources are abundant.

## Signal transduction hub

**Fig. 2:**
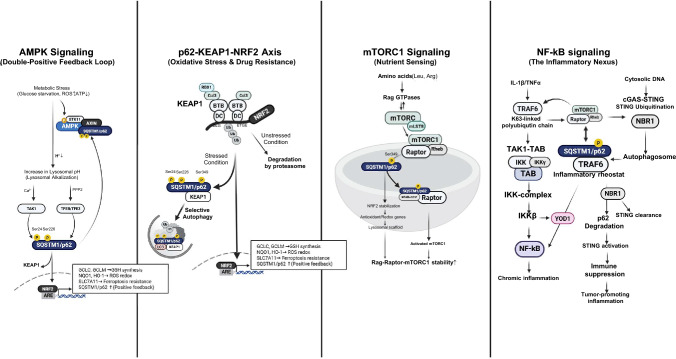
SQSTM1/p62 as a multimodal signal transduction hub orchestrating oncogenic fitness. The diagram depicts p62 as a central molecular switchboard that integrates diverse oncogenic and homeostatic pathways to translate proteostatic and metabolic status into coherent survival outputs. Within the NRF2-KEAP1 axis, site-specific phosphorylation of p62—particularly at Ser349, as well as Ser24 and Ser226—facilitates the competitive sequestration and subsequent autophagic degradation of KEAP1, which stabilizes NRF2 and induces a robust antioxidant response element (ARE)-driven transcriptional program. Concurrently, p62 acts as an indispensable scaffold for AMPK signaling under metabolic stress; phosphorylation at Ser24 and Ser226 by TAK1 is required for the recruitment of the AXIN-STK11 (LKB1)-AMPK complex to the lysosomal surface, thereby activating the lysosomal AMPK pool. Beyond energy sensing, p62 coordinates anabolic growth by scaffolding Rag GTPases and Raptor for mTORC1 activation, while simultaneously promoting pro-inflammatory signaling through TRAF6-mediated NF-κB activation. Finally, p62 exerts bimodal control over the cGAS-STING axis by either attenuating innate immunity through autophagic STING clearance or fostering a pro-tumorigenic inflammatory niche. Together, these integrated p62-centered signaling nodes confer the metabolic plasticity and adaptive resilience essential for tumor progression and therapy resistance

P62 functions as a multimodal signal integrator, orchestrating inputs from major oncogenic and homeostatic pathways: AMPK, NRF2, mTORC1, NF-κB, and cGAS-STING. Its capacity to serve as a physical scaffold enables the spatiotemporal coordination of these often disparate signals, effectively translating proteostatic status into specific cellular outputs (Fig. 2).

### The p62-KEAP1-NRF2 axis: oxidative stress and NRF2 addiction in cancer

SQSTM1/p62 is a well-characterized direct transcriptional target of NRF2, driven by antioxidant response element (ARE) sequences in the SQSTM1 promoter [70]. As a result, elevated p62 levels frequently reflect prior NRF2 activation rather than its cause. Under basal conditions, NRF2 is maintained at minimal levels by KEAP1, which facilitates NRF2 ubiquitination via the Cul3–Rbx1 E3 ligase complex through a "two-site latch" model involving the high-affinity ETGE and low-affinity DLG motifs [71, 72]. Critically, however, p62 is not merely a passive downstream reporter of NRF2 activity but also functions as an essential upstream activator [14]. SQSTM1 knockout cells exhibit significantly attenuated NRF2 activation in response to both metabolic stress and oxidative stress, demonstrating that p62 is required for full NRF2 induction under stress conditions [39, 47, 73]. This upstream activation operates through a multiple phosphorylation-dependent mechanism. The KEAP1-Interacting Region (KIR) of p62 structurally mimics the ETGE motif, yet basal p62–KEAP1 affinity remains low. Phosphorylation of p62 at Ser349 (Ser351 in mice) dramatically enhances this interaction, enabling p62 to competitively sequester KEAP1 into autophagic condensates and promote its lysosomal degradation [31]. More recently, phosphorylation of p62 at Ser24 and Ser226—triggered by either metabolic and oxidative stress—has been identified as an additional layer of NRF2 activation [47]. These modifications facilitate the sequestration of KEAP1, promoting its autophagic degradation independently of canonical oxidative cysteine modifications on KEAP1. This stabilizes NRF2, drives its nuclear translocation, and activates an ARE-dependent transcriptional program—including the re-induction of SQSTM1 itself, thereby completing a self-reinforcing positive feedback loop. In cancer, constitutive engagement of this loop drives "NRF2 addiction," conferring resistance to ROS-generating therapies and ferroptosis in hepatocellular carcinoma (HCC) and lung adenocarcinoma [18, 74, 75].

### AMPK signaling: the double-positive feedback loop

Historically, AMPK and growth signaling were viewed as antagonistic pathways, but p62 has redefined this relationship as a synergistic energy-sensing mechanism during metabolic crises. Pioneering studies established that the AXIN-STK11 complex assembles at the lysosomal membrane in response to glucose deprivation, providing a spatial platform for AMPK activation [76–79]. Building on this framework, our recent study demonstrated that TAK1-mediated phosphorylation of p62 at Ser24 and Ser226 is essential for recruiting and stabilizing the AXIN-STK11 complex at the lysosomal membrane, thereby enabling lysosomal AMPK activation [47]. Once activated, AMPK promotes SQSTM1 transcription via the TFEB/TFE3 axis, establishing a context-dependent double-positive feedback loop wherein AMPK induces p62, and p62 in turn sustains AMPK activation. It is important to note that this p62-scaffolded lysosomal AMPK activation mechanism has been most comprehensively characterized under glucose deprivation conditions in lung cancer cells. Critically, this pathway may exhibit temporal specificity: the AXIN-STK11 complex operates primarily during early-phase metabolic stress when glucose metabolism is impaired but cellular ATP levels remain relatively preserved, serving as an early-warning nutrient stress sensor [80]. In contrast, during late-stage metabolic stress characterized by profound ATP depletion and elevated AMP/ATP ratios, AMPK activation proceeds predominantly through the canonical AMP/ATP ratio-sensing mechanism via direct STK11-mediated phosphorylation of AMPK, independent of the AXIN-SQSTM1 lysosomal scaffold. This temporal distinction suggests that p62-dependent AMPK activation enables proactive metabolic adaptation prior to energetic crisis, while other AMPK regulatory inputs (AMP/ATP ratio, CaMKK2 signaling) may dominate in different metabolic contexts or stress stages.

### mTORC1 signaling: coupling nutrient sensing to anabolic drive

As a downstream integrator of nutrient availability, p62 serves as a core component of the lysosomal amino acid-sensing machinery. By interacting with Raptor and Rag GTPases on the lysosomal surface, p62 stabilizes the active Rag heterodimer under nutrient-replete conditions. This facilitates the recruitment and subsequent activation of mTORC1 by Rheb [23, 24]. Furthermore, the mTORC1-mediated phosphorylation of p62 at Ser349 creates a specialized anabolic-antioxidant feedback loop. While this phosphorylation activates the NRF2 pathway as discussed in "Glucose metabolism and the orchestration of the Warburg phenotype", it simultaneously reinforces the scaffolding role of p62 in nutrient sensing, thereby coupling the anabolic drive of the cell with robust redox protection [81, 82].

### NF-κB and cGAS-STING: inflammatory and immune coordination

P62 also acts as an essential scaffold for the canonical activation of NF-κB in response to pro-inflammatory cytokines like IL-1β and TNFα. By engaging the E3 ligase TRAF6 via its TB domain, p62 promotes K63-linked polyubiquitination, which serves as a scaffold for the TAK1/TAB and IKK complexes. This culminates in IKKβ phosphorylation and the nuclear translocation of p65/p50 [83–85]. The persistence of this signaling is regulated by the deubiquitinase YOD1, which acts as a molecular brake. In cancer, the downregulation of YOD1 allows for unchecked p62-TRAF6 interactions, fueling chronic inflammation [70].

Finally, p62 exerts bimodal control over the cGAS-STING innate immune pathway. While it typically attenuates STING signaling by delivering ubiquitinated STING to autophagosomes for degradation [86], it can paradoxically promote STING activation in specific microenvironments like the HCC stroma. By facilitating TRIM32-mediated ubiquitination of STING, p62 promotes a pro-tumorigenic inflammatory niche, where the balance between p62-driven activation and NBR1-driven degradation determines the immune status of the tumor [76].

## Metabolic reprogramming: the p62-centric wiring of cancer metabolism

**Fig. 3:**
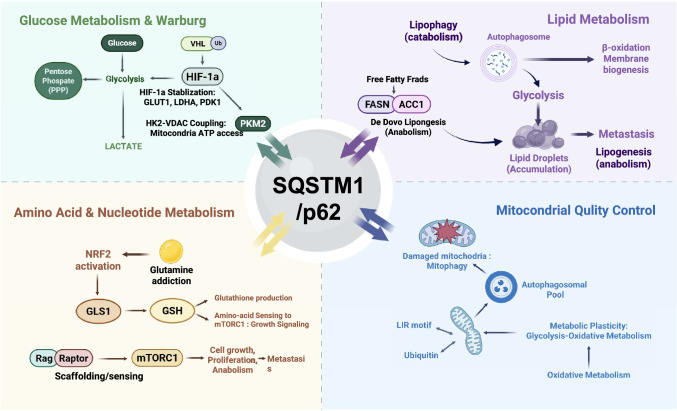
Context-dependent oncogenic adaptation orchestrated by p62 across diverse tumor types. The schematic summarizes the tissue-specific and genotype-dependent manifestations of p62-mediated signaling and metabolic reprogramming, reflecting a functional state positively selected for tumor survival. In Hepatocellular Carcinoma (HCC), p62-driven NRF2 addiction within Mallory-Denk bodies confers redox defense and sorafenib resistance. In Non-Small Cell Lung Cancer (NSCLC), p62 supports metabolic fitness in *STK11/LKB1*- or *KEAP1*-mutant backgrounds by providing an alternative stress kinase interface for AMPK activation. The p62-centric wiring further facilitates lineage plasticity in prostate cancer through androgen receptor (AR) turnover and neuroendocrine differentiation, while in Glioblastoma (GBM), nuclear p62 compromises DNA repair capacity by interfering with chromatin remodeling. Finally, the persistence of p62/NBR1 condensates supports the metastatic cascade by stabilizing EMT-associated transcription factors and remodeling the cytoskeletal architecture. Collectively, these organ-specific programs illustrate how p62 enables malignant cells to navigate distinct selective pressures within the tumor microenvironment

The metabolic landscapes of cancer cells are shaped by stringent evolutionary selection within the tumor microenvironment, favoring the emergence of rewired networks that sustain rapid proliferation and survival under nutrient-constricted and hypoxic conditions. Within this selective framework, p62 occupies a pivotal position as a metabolic rheostat that integrates selective autophagy, nutrient sensing, and stress-responsive transcriptional programs. By acting as a multifaceted molecular scaffold, p62 facilitates the flexible transition between anabolic and catabolic states—a trait positively selected to underpin oncogenic metabolic plasticity (Fig. 3) (Table 2).

**Table 2:** p62-driven metabolic reprogramming: summary of "Metabolic reprogramming: the p62-centric wiring of cancer metabolism"

Metabolic node	p62-linked mechanism	Dominant functional output	Representative cancer contexts	Experimental validation approach		In vivo evidence	References
Glucose metabolism (Warburg phenotype)	Interference with VHL E3 ligase → HIF-1α stabilization	Enhanced glucose uptake, aerobic glycolysis, lactate production	NSCLC, HCC, glioblastoma	SQSTM1 knockdown; rescue assays; HIF1α stability assay		Xenograft tumor growth models	Chen et al., *J Cell Sci* 2016 [87]
Glycolytic branching/PPP	Bias toward low-activity PKM2 state; NRF2-dependent induction of PPP enzymes (G6PD, PGD, TKT)	NADPH production; ribose-5-phosphate synthesis	NRF2-activated solid tumors	SQSTM1 knockdown; KEAP1-binding mutants; NRF2 transcriptional assays		Liver tumor models	Sun et al., *Hepatology* 2016 [75]
Lipid droplets (lipophagy)	Recognition of ubiquitinated LD surface proteins (PLIN2) and recruitment of autophagosomes	Mobilization of fatty acids for β-oxidation	ccRCC; prostate cancer	p62 expression; LC3 recruitment assays		Mouse liver models	Yang et al., *Genes Dev* 2011 [128]
De novo lipogenesis	Stabilization or selective turnover of lipogenic enzymes (FASN, ACC1)	Maintenance of lipid supply	Prostate cancer	SQSTM1 Knockout, deletion	(C289,290S); enzyme stability assays	Xenograft models	Huang et al., *Mol Cell* 2023 [92]
Amino acid metabolism (glutamine)	NRF2-driven induction of SLC1A5 and GLS1	Anaplerosis; glutathione synthesis	KRAS-driven PDAC	Genetic deletion; NRF2 activity assays		GEMM PDAC models	Lam et al., *Cancer Res* 2017 [98]; Mukhopadhyay et al., *Cancer Res* 2020 [96]
mTORC1 nutrient sensing	Scaffolding of Rag GTPases and Raptor at lysosomes	Coupling amino acid availability to anabolic growth	Multiple solid tumors	SQSTM1 knockdown; Rag interaction assays		Mouse models	Duran et al., *Mol Cell* 2011 [23]; Linares et al., *Mol Cell* 2013 [24]
Nucleotide metabolism (PPP flux)	NRF2-mediated upregulation of G6PD and related enzymes	dNTP pool maintenance; replication stress tolerance	Highly proliferative cancers	SQSTM1 knockdown; NRF2 target gene analysis		Liver cancer models	Sayin et al., *eLife* 2017 [99]
Mitochondrial quality control (mitophagy)	UBA-mediated capture of ubiquitinated mitochondria and LC3 recruitment	Removal of ROS-producing mitochondria; metabolic adaptability	Metastatic and therapy-stressed tumors	SQSTM1 KO; Parkin-mediated mitophagy assays		Xenograft stress models	Ma et al., *iScience* 2020 [100]

### Glucose metabolism and the orchestration of the Warburg Phenotype

Aggressive malignancies frequently favor aerobic glycolysis to meet their biosynthetic demands, and p62 reinforces this "Warburg phenotype" through several distinct entry points. A key mechanism involves the stabilization of HIF-1α; p62 has been reported to interface with the VHL E3 ligase machinery, thereby reducing the ubiquitination and subsequent degradation of HIF-1α. The resulting stabilization of HIF-1α facilitates the upregulation of glucose transporters (e.g., GLUT1) and glycolytic enzymes (e.g., LDHA, PDK1), ensuring robust glucose uptake and lactate production even under normoxic conditions [87].

Furthermore, p62 coordinates the mitochondrial positioning and stability of Hexokinase 2 (HK2), promoting its association with VDAC on the outer mitochondrial membrane. This configuration grants HK2 privileged access to mitochondrial ATP for glucose phosphorylation while simultaneously blunting apoptosis by stabilizing the mitochondrial membrane potential. In certain contexts, this HK2–VDAC coupling is further sustained by p62-mediated scaffolding of upstream survival signaling, such as the AKT pathway [88]. Additionally, p62 can modulate glycolytic branching by regulating PKM2 abundance/availability through SQSTM1-dependent autophagy [89]. Reduced PKM2 catalytic activity whether through lowered PKM2 levels or inhibitory modifications can create a bottleneck at the final step of glycolysis, promoting diversion of upstream intermediates into anabolic shunt pathways such as the pentose phosphate pathway (PPP), to support NADPH generation and biomass production [90].

### Lipid metabolism and systemic energy homeostasis: from lipophagy to adipose tissue regulation

Lipids are indispensable for membrane biogenesis, intracellular signaling, and energy storage. p62 governs lipid homeostasis by balancing catabolic mobilization with anabolic synthesis in a context-dependent manner. As a selective autophagy receptor, p62 facilitates lipophagy by recognizing ubiquitinated proteins on the surface of lipid droplets, such as PLIN2/ADRP, and recruiting autophagosomes to mobilize stored triglycerides [91]. The resulting lysosomal breakdown releases free fatty acids that fuel mitochondrial β-oxidation or support membrane synthesis during periods of nutrient deprivation.

Conversely, p62 is also intimately involved in maintaining de novo lipogenic capacity. In several tumor contexts, p62 has been shown to stabilize lipogenic enzymes, including Fatty Acid Synthase (FASN), protecting them from premature turnover. Interestingly, recent observations suggest an autophagy-linked checkpoint involving ACC1; perturbation of ACC1 can trigger p62-dependent targeting of the lipogenic machinery for selective degradation. This feedback logic ensures that lipid-synthesis enzymes themselves become regulated "cargo" when lipid homeostasis is disrupted. In aggressive tumors such as clear cell renal cell carcinoma (ccRCC) and prostate cancer, high p62 expression often coincides with an increased lipid droplet burden, providing an energy reservoir that facilitates migration and metastatic outgrowth [92].

Notably, systemic Sqstm1 knockout mice develop mature-onset obesity, leptin resistance, and insulin intolerance, with significantly reduced metabolic rate [93, 94]. Mechanistically, loss of p62 leads to enhanced basal ERK activity in adipose tissue, promoting adipogenesis through increased PPAR-γ and decreased UCP-1 expression. p62 normally sequesters ERK and inhibits its nuclear translocation; loss of this inhibitory function results in constitutive ERK activation and accelerated adipocyte differentiation. Tissue-specific knockout studies further revealed that adipocyte-specific p62 deficiency causes obesity due to impaired nonshivering thermogenesis and mitochondrial dysfunction in brown adipose tissue, where p62 is required for β-adrenergic stimulation via p38 MAPK signaling [95]. These findings establish dual roles for p62 in white adipose tissue adipogenesis (via ERK inhibition) and brown adipose tissue thermogenesis (via p38-PGC1α-UCP1 axis). Further, our recent findings demonstrating that p62 is essential for AMPK activation under metabolic stress provide an additional mechanistic layer, suggesting that impaired p62-AMPK signaling may further contribute to reduced fatty acid oxidation and energy expenditure—a hypothesis that warrants future experimental validation [47]. Understanding these tissue-specific physiological roles is critical for developing safe p62-targeting therapeutics that selectively disrupt tumor-promoting functions while preserving normal metabolic homeostasis.

### Amino acid metabolism and the glutamine-NRF2 axis

The integration of amino acid sensing and utilization is another hallmark of p62-driven metabolic wiring. In oncogenic settings, particularly Ras-driven models, the p62-NRF2 signaling axis enhances glutamine addiction by upregulating transporters such as SLC1A5 and enzymes like GLS1 [96–98]. This redirection of glutamine flux supports anaplerosis to replenish TCA cycle intermediates and provides the precursors necessary for glutathione (GSH) synthesis, thereby bolstering the cell’s antioxidant defenses [96, 99].

Beyond transcriptional regulation, p62 participates directly in the physical amino-acid–sensing apparatus at the lysosome. Through its interactions with Raptor and the Rag GTPase system, p62 couples the immediate availability of amino acids to the activation of mTORC1 [23]. This dual role positions p62 as both a metabolic effector that drives glutamine utilization and a nutrient sensor that gatekeeps anabolic growth signaling [98].

### Nucleotide Metabolism and PPP Flux for Genome Maintenance

Through its synergistic relationship with NRF2, p62 biases glucose-derived carbons toward the Pentose Phosphate Pathway (PPP) by increasing the expression of rate-limiting enzymes such as G6PD, PGD, and TKT. This metabolic redirection serves two critical functions: (i) the generation of ribose-5-phosphate for de novo nucleotide synthesis and (ii) the production of NADPH for redox buffering. By sustaining robust NADPH and dNTP pools, p62-centered metabolic wiring indirectly shields proliferating cancer cells from replication stress and DNA damage, thereby promoting genome maintenance and survival under therapeutic pressure [75].

### Mitochondrial quality control and metabolic plasticity

Maintaining mitochondrial integrity is essential for tumor adaptability. In the canonical PINK1/Parkin pathway, damaged or dysfunctional mitochondria are ubiquitinated and subsequently captured by p62 via its UBA domain. Through its LIR motif, p62 links these damaged organelles to autophagosomes for clearance (mitophagy) [100]. By removing mitochondria that are prone to ROS leakage and cytochrome *c* release, p62 ensures a healthy mitochondrial pool. This quality control mechanism is vital for metabolic plasticity, enabling cancer cells to switch efficiently between glycolysis and oxidative phosphorylation during the stressful transitions of tumor dissemination and therapy resistance.

## p62 in cancer

### Pan-cancer overexpression and prognostic value

The convergence of scaffolded signaling, post-translational regulation, and metabolic control positions SQSTM1/p62 as far more than a passive readout of impaired proteostasis. Integrating large-scale genomic and proteomic repositories reveals that p62 is aberrantly upregulated across a diverse spectrum of malignancies, including non-small cell lung cancer (NSCLC), hepatocellular carcinoma (HCC), pancreatic ductal adenocarcinoma (PDAC), breast cancer, and glioblastoma [101, 102]. In many patient cohorts, high p62 expression serves as a contextual biomarker that correlates with adverse clinicopathologic features such as advanced tumor grade, increased therapy resistance, and a higher propensity for metastasis [87, 88]. The prognostic utility of p62 is particularly enhanced when integrated with specific genomic markers; for instance, in gliomas, combining p62 levels with IDH mutation status provides superior prognostic stratification compared to either marker alone [19]. Similarly, in breast cancer, p62 accumulation is significantly linked to aggressive phenotypes, including HER2-positive and triple-negative subtypes, reflecting a role in maintaining stress-tolerant phenotypes [103, 104]. Mechanistically, this prognostic power stems from p62’s capacity to coordinate multiple stress-response outputs simultaneously—ranging from redox buffering via the KEAP1–NRF2 axis to survival signaling through TRAF6–NF-κB and mTORC1-mediated growth control [16, 105–107]. These diverse outputs are intricately tuned by site-specific post-translational modifications (PTMs), which dictate whether p62 stabilizes signaling-competent assemblies or routes cargo toward degradative pathways. The pan-cancer landscape of p62 and organ-specific mechanisms that exemplify how this protein rewires growth, redox balance, immune interactions, and metastatic competence are summarized in Table 3.

**Table 3:** The role of p62 in the cancer context: summary of "p62 in Cancer"

Cancer context	Dominant p62-associated axis	Functional outcome	Genetic/functional validation approach	In vivo tumor model evidence	References
Pan-cancer (multiple solid tumors)	NRF2, NF-κB, mTORC1	Stress tolerance; therapy resistance	SQSTM1 knockdown; correlation with NRF2 target genes	Reported in xenograft systems	Ling et al., *Cancer Cell* 2012 [17]; Umemura et al., *Cancer Cell* 2016 [18]
Hepatocellular carcinoma (HCC)	KEAP1–NRF2; mTORC1; cGAS–STING	Redox buffering; hepatocarcinogenesis	Sqstm1 genetic deletion; KEAP1 binding mutants	Chemical and genetic HCC models show ↓ tumor burden in Sqstm1 KO	Umemura et al., *Cancer Cell* 2016 [18]; Sun et al., *Hepatology* 2016 [75]
NSCLC (KEAP1 mutation)	Constitutive NRF2 activation	Metabolic adaptation; therapy resistance	CRISPR-mediated SQSTM1 deletion; KEAP1-deficient models	Xenograft growth suppression upon SQSTM1 depletion	Romero et al., *Cancer Discov* 2017 [131]
NSCLC (STK11/LKB1 loss)	p62–TAK1–AMPK scaffolding	Metabolic stress tolerance	S24A/S226A phospho-deficient mutants; CRISPR KO	Xenograft models with reduced tumor growth	Choi et al., *Autophagy* 2024 [47]
SQSTM1–ALK fusion NSCLC	PB1-mediated oligomerization	Ligand-independent ALK signaling	Fusion protein expression analysis	Tumorigenesis in model systems	Relevant fusion studies
Pancreatic ductal adenocarcinoma (PDAC)	Autophagy–NRF2 coupling	Tumor maintenance in nutrient-poor microenvironment	SQSTM1 knockdown; Genetic deletion S235/236; NRF2 pathway modulation	GEMM PDAC models show reduced tumor development upon Sqstm1 disruption	Ling et al., *Cancer Cell* 2012 [17]; Lam et al., *Cancer Res* 2017 [98]
Prostate cancer (PRAD)	AR turnover; NF-κB; metabolic rewiring	Castration resistance; lineage plasticity	p62 depletion; AR stability assays; NF-κB reporter assays	Metastasis and xenograft models	Clark et al., *Cancer Lett* 2024 [130]
Glioblastoma (GBM)	YAP/TAZ; nuclear p62–DDR axis	Stemness; radioresistance	Localization mutants; DNA damage assays	Orthotopic xenograft sensitivity to radiation upon p62 modulation	Wang et al., *Autophagy* 2024 [129]
Metastatic programs (EMT)	Stabilization of EMT-TFs; condensate formation	Migration and invasion	p62 knockdown; EMT marker analysis	Mouse metastasis models	Li et al., *Carcinogenesis* 2017 [113]
Vascular smooth muscle cells (non-cancer control)	NF-κB modulation	Increased proliferation/migration in SQSTM1-deficient cells	SQSTM1 genetic deletion	In vitro system (non-tumor context)	

### Context-dependent oncogenic functions of p62 and the p62–NRF2 axis

Across a wide array of tumor types, elevated p62 levels frequently represent a functional state that actively supports survival under oxidative, nutrient, and therapeutic stress. While this conclusion is supported by a substantial body of experimental evidence, an important methodological caveat warrants consideration: most mechanistic studies are conducted under supraphysiological oxygen tension (~ 18–21%), which may artificially elevate basal NRF2 activity and thereby confound the interpretation of p62 function. However, extensive in vivo evidence from GEMMs and xenograft models—where oxygen tension, metabolic stress, and stromal interactions are physiologically relevant—consistently demonstrates that Sqstm1 depletion significantly reduces tumor burden across hepatocellular carcinoma [18, 108], pancreatic cancer [17], and other malignancies [109, 110]. These findings, derived from physiological tumor microenvironments with hypoxic or normoxic oxygen levels, confirm p62's functional requirement for tumor growth beyond in vitro culture artifacts and validate its relevance as a therapeutic target.

However, it is critical to recognize that the relationship between p62 and NRF2 exhibits context-dependent directionality across cancer types. In cancers harboring KEAP1 loss-of-function (e.g., lung adenocarcinoma) or NFE2L2 (NRF2) gain-of-function mutations (e.g., lung squamous cell carcinoma), elevated p62 expression is frequently a secondary consequence of constitutive NRF2 activation, with p62 serving as a biomarker of NRF2 pathway hyperactivation rather than its primary driver [111, 112]. This tissue-specific difference underscores the importance of genetic and metabolic context in determining whether p62 functions downstream or upstream of NRF2 in different cancers.

### Metastasis, EMT, and the physical state of p62 assemblies

The metastatic cascade, a primary driver of cancer mortality, is heavily influenced by p62 through its interaction with the epithelial–mesenchymal transition (EMT) machinery. p62 has been shown to stabilize EMT-associated transcription factors such as TWIST1 and SNAIL, thereby sustaining programs that repress epithelial identity and promote motility. Additionally, p62 interfaces with the cytoskeletal architecture, specifically intermediate filaments like vimentin, to facilitate the structural reorganization required for cellular migration and invasion [113, 114].

Emerging work adds a critical biophysical dimension to these observations, proposing that the material state of p62 assemblies dictates metastatic competence. p62/NBR1-driven condensates have been implicated in promoting metastasis by sequestering negative regulators of migration, suggesting that the persistence and biophysical properties of these bodies—rather than their mere presence—determine oncogenic output [115]. This concept provides a unifying framework to reconcile how p62 can support both degradative functions and long-lived signaling behaviors, depending on how condensates are physically organized and regulated by the PTM code.

## Therapeutic targeting and future directions

Given its central role as a multimodal scaffold integrating signaling, phosphorylation, and metabolism, SQSTM1/p62 represents an appealing but non-conventional therapeutic target. Unlike traditional drug targets such as kinases or proteases, p62 lacks a catalytic active site. This structural characteristic necessitates a paradigm shift in therapeutic strategy, moving away from direct enzymatic inhibition toward the nuanced modulation of protein–protein interactions (PPIs), higher-order oligomerization, and cargo recruitment.

Recent pharmacological advances have demonstrated that p62 activity can be perturbed indirectly by altering its structural state or interaction interfaces. For instance, the FDA-approved photosensitizer Verteporfin has been found to promote the covalent cross-linking of p62 into insoluble, high-molecular-weight assemblies, effectively sequestering it from its signaling partners, including NRF2 and the YAP/STAT3 pathways [116]. Early clinical observations in glioblastoma and prostate cancer suggest that such interference with p62 assemblies can effectively sensitize tumors to standard-of-care therapies. More selective approaches are also emerging; compounds such as XRK3F2 target the p62 ZZ domain to disrupt stress-adaptive signaling [117, 118], while additional candidates specifically designed to block the p62–KEAP1 interface illustrate the feasibility of domain-specific intervention [82, 119].

While p62 represents a compelling therapeutic target, critical limitations must be addressed. First, p62 is essential for proteostasis in normal tissues, as its genetic ablation in mice leads to a broad spectrum of pathological phenotypes—including mature-onset obesity, leptin resistance, and systemic insulin resistance [93]; Paget's disease-like bone lesions driven by enhanced osteoclastogenesis [120]; exacerbated neurodegeneration with accumulation of hyperphosphorylated tau and neurofibrillary tangles [121]; and accelerated cellular senescence in vascular tissues [122, 123]. However, a therapeutic window may exist between cancer cells exhibiting p62 addiction and normal tissues retaining redundancy [124, 125]. Second, p62 dependency varies across cancer types. Tumors with KEAP1 mutations, autophagy deficiency, or metabolic stress may show exceptional vulnerability, necessitating companion diagnostics for patient stratification [126]. Thus, comprehensive preclinical studies will be essential to identify optimal patient populations for clinical translation. Third, combination strategies with metabolic stress inducers, pro-oxidant therapies, or autophagy modulators may offer superior efficacy while widening therapeutic windows [127]. Future progress in the field will likely depend on precision strategies that account for specific tumor contexts, distinct phosphorylation states—such as the Ser24/Ser226 axis identified in our research—and particular pathway dependencies, rather than the global suppression of p62 function.
